# Amiodarone for arrhythmia in patients with Chagas disease: A systematic review and individual patient data meta-analysis

**DOI:** 10.1371/journal.pntd.0006742

**Published:** 2018-08-20

**Authors:** Cinara Stein, Celina Borges Migliavaca, Verônica Colpani, Priscila Raupp da Rosa, Daniel Sganzerla, Natalia Elis Giordani, Sandro Renê Pinto de Sousa Miguel, Luciane Nascimento Cruz, Carisi Anne Polanczyk, Antonio Luiz P. Ribeiro, Maicon Falavigna

**Affiliations:** 1 Institute for Education and Research, Hospital Moinhos de Vento, Porto Alegre, Brazil; 2 Federal University of Rio Grande do Sul, Porto Alegre, Brazil; 3 Centro Universitário FADERGS, Porto Alegre, Brazil; 4 Faculdade Meridional–IMED, Passo Fundo, Brasil; 5 National Institute of Science and Technology for Health Technology Assessment, Post-Graduate Program of Epidemiology, Federal University of Rio Grande do Sul, Porto Alegre, Brazil; 6 Hospital das Clinicas and School of Medicine, Universidade Federal de Minas Gerais, Belo Horizonte, Brazil; 7 Department of Health Research Methods, Evidence and Impact, McMaster University Health Sciences Centre, Hamilton, Canada; Center for Biologics Evaluation and Research, Food and Drug Administration, UNITED STATES

## Abstract

**Background:**

Chagas disease is a neglected chronic condition caused by *Trypanosoma cruzi*, with high prevalence and burden in Latin America. Ventricular arrhythmias are common in patients with Chagas cardiomyopathy, and amiodarone has been widely used for this purpose. The aim of our study was to assess the effect of amiodarone in patients with Chagas cardiomyopathy.

**Methodology:**

We searched MEDLINE, Embase and LILACS up to January 2018. Data from randomized and observational studies evaluating amiodarone use in Chagas cardiomyopathy were included. Two reviewers selected the studies, extracted data and assessed risk of bias. Overall quality of evidence was accessed using Grading of Recommendations Assessment, Development and Evaluation (GRADE).

**Principal findings:**

We included 9 studies (3 before-after studies, 5 case series and 1 randomized controlled trial). Two studies with a total of 38 patients had the full dataset, allowing individual patient data (IPD) analysis. In 24-hour Holter, amiodarone reduced the number of ventricular tachycardia episodes in 99.9% (95%CI 99.8%-100%), ventricular premature beats in 93.1% (95%CI 82%-97.4%) and the incidence of ventricular couplets in 79% (RR 0.21, 95%CI 0.11–0.39). Studies not included in the IPD analysis showed a reduction of ventricular premature beats (5 studies), ventricular tachycardia (6 studies) and ventricular couplets (1 study). We pooled the incidence of adverse side effects with random effects meta-analysis; amiodarone was associated with corneal microdeposits (61.1%, 95%CI 19.0–91.3, 5 studies), gastrointestinal events (16.1%, 95%CI 6.61–34.2, 3 studies), sinus bradycardia (12.7%, 95%CI 3.71–35.5, 6 studies), dermatological events (10.6%, 95%CI 4.77–21.9, 3 studies) and drug discontinuation (7.68%, 95%CI 4.17–13.7, 5 studies). Quality of evidence ranged from moderate to very low.

**Conclusions:**

Amiodarone is effective in reducing ventricular arrhythmias, but there is no evidence for hard endpoints (sudden death, hospitalization). Although our findings support the use of amiodarone, it is important to balance the potential benefits and harms at the individual level for decision-making.

## Author summary

Chagas disease is a chronic neglected tropical disease, with high prevalence and burden in Latin America. About 30% of chronically infected patients develop Chagas cardiomyopathy. Ventricular arrhythmias are common in patients with Chagas cardiomyopathy and treatment approaches include medications, resynchronization therapy, and implantable cardioverter defibrillator. Studies published from 1980 to 1990 have evaluated the effect of amiodarone. According to our systematic review and individual patient meta-analysis, amiodarone reduced ventricular tachycardia, ventricular premature beats and incidence of ventricular couplets. Although the strong evidence of clinical benefit with arrhythmia reduction, this information should be interpreted with caution, since arrhythmia is a surrogate outcome and since its clinical impact on death and hospitalization reduction over time is not clear. Little information was identified related to hard endpoints. Regarding side effects, our systematic review observed that amiodarone was associated with corneal microdeposits, gastrointestinal events, sinus bradycardia, dermatological events, pneumonitis, hypothyroidism and drug discontinuation. The currently available evidence shows that amiodarone seems to be an effective antiarrhythmic drug for patients with Chagas disease, especially in settings where an implantable cardioverter defibrillator is not available or affordable, but that a balance between potential benefits and harms at the individual level is needed.

## Introduction

Chagas disease, also known as American trypanosomiasis [1], is an important public health problem in Latin America, and remains a substantial cause of morbidity and mortality [2]. Recently, it has become a health concern [1] in areas such as Europe, North America, Japan and Australia, especially due to immigration from endemic areas to developed countries [3,4]. About 6 to 8 million people are affected worldwide [5], and less than 1% of infected people have access to adequate diagnosis and treatment [6].

The clinical course of the disease is extremely variable, and although many individuals remain asymptomatic for long periods, approximately 30% of infected people develop Chagas cardiomyopathy [7]. Cardiac complications result in remodeling of the cardiac collagenous matrix and subsequent fibrosis, leading to increased myocardial stiffness, systolic and diastolic dysfunction, and ultimately a severe dilated cardiomyopathy associated with ventricular arrhythmias [8].

Since trypanocidal therapy with benznidazole in patients with established Chagas cardiomyopathy had not proved to reduce cardiac clinical deterioration in clinical trials [9], the treatment of heart failure and arrhythmias became the main therapeutic strategy to curb the evolution of the disease. Despite the lack of solid evidence-based data on Chagas disease, therapy should generally be instituted extrapolating from guidelines for the management of heart failure patients, including neurohumoral inhibition, resynchronization therapy, and implantable cardioverter defibrillator [10]. However, this alternative is not readily available for low income populations such as those affected by Chagas disease. Studies have shown that amiodarone can improve survival in patients who have a high risk of arrhythmic death [11,12]; thus, amiodarone has been recommended as the treatment of choice for all patients with sustained ventricular tachycardia, and for those with no sustained ventricular tachycardia and myocardial dysfunction [13]. In a recent meta-analysis of primary prevention (17 studies, 8383 participants), amiodarone reduced sudden death, cardiac mortality and all-cause mortality; however, Chagas patients were not included. Nevertheless, amiodarone was associated with increased adverse effects, both hypo- or hyperthyroidism and pulmonary fibrosis, and increased risk of discontinuation compared with placebo [14].

Due to the lack of direct evidence regarding the benefits and risks of amiodarone use in Chagas patients, evidence from patients with other cardiopathies is often used and extrapolated to patients with Chagas disease for decision-making. However, there are some particularities of the amiodarone effect on Chagas disease; for instance, there is some evidence that amiodarone also has anti-*T*. *cruzi* activity, disrupting Ca2+ homeostasis and blocking oxidosqualene cyclase in *T*. *cruzi*, causing ultrastructural damage [15]. Only small studies have assessed the effects of amiodarone specifically in Chagas disease, mostly published in Portuguese and Spanish, and it is not clear if the estimated effect is similar to that in the general population. Therefore, our aim was to do a systematic review of the effect of amiodarone in patients with Chagas disease, to provide a more accurate estimate of the effect of amiodarone in this population and to identify the potential benefits and harms of this drug.

## Methods

### Protocol and registration

The planning for this review was based on the guidelines of Preferred Reporting Items for Systematic Reviews and Meta-analyses (PRISMA) [16] and in accordance with the Cochrane Handbook for Systematic Reviews of Interventions [17]. This systematic review was registered in PROSPERO under No. CRD42017056765 (S1 Appendix).

### Eligibility criteria and outcomes of interest

We included clinical trials, crossover studies, case series and before-after studies assessing the effects of amiodarone on symptoms in patients with Chagas disease. Case-control studies, reviews, letters and editorials were not included. The outcomes assessed were any arrhythmia (e.g., ventricular tachycardia, ventricular premature beats and ventricular couplets), sudden death and side effects.

### Search strategy

The following electronic databases were searched: MEDLINE (accessed through PubMed), Embase and LILACS. In addition, the references included in the published articles identified were used as an additional source to identify other studies. The search was conducted in January 2018 and consisted of the following terms: “Chagas disease” and “amiodarone,” as shown in S2 Appendix. There was no language restriction in the search.

### Study selection and data extraction

The titles and abstracts of all articles identified by the search strategy were evaluated by two researchers (C.S., C.B.M.). All abstracts that did not provide sufficient information concerning the inclusion and exclusion criteria were selected to evaluate the full article. In the second phase, the same reviewers independently evaluated the full articles and made their selection in accordance with the eligibility criteria. Disagreements between reviewers were resolved by consensus or by a third reviewer (V.C.).

Data extraction was performed using a standardized form by two reviewers independently (C.S., C.B.M.). The following data were extracted: methodological characteristics of the studies, number of participants, comparison groups, interventions and outcomes. Extracted outcomes were arrhythmia, side effects and sudden death. The authors were contacted by email if full data were not available. Disagreements between reviewers were resolved by consensus or by a third reviewer (V.C.).

### Quality assessment

The assessment of methodological quality was performed by two researchers (C.S, C.B.M.). The clinical and crossover studies were evaluated using the RoB 2.0 tool to assess risk of bias in randomized trials and crossover studies [18], and the before-after studies were evaluated using the NIH Quality Assessment Tool for Before–After Studies with No Control Group [19]. For case series, we used the same tool for before-after studies, considering only the applicable questions.

The overall quality of evidence was assessed using Grading of Recommendations Assessment, Development and Evaluation (GRADE). GRADE is a system used for grading the quality of evidence, classifying each outcome as high, moderate, low, or very low, corresponding to the level of certainty on the estimate results. [20]. We created an Evidence Profile and Summary of Findings Tables for each outcome using GRADE’s electronic tool GRADEpro GDT (www.gradepro.org).

### Data synthesis and analysis

After data extraction, if the outcome measurements could not be transformed in a common numeric scale for quantitative synthesis due to different designs of trials, a descriptive synthesis was performed.

Individual patient data (IPD) meta-analysis was performed using Poisson and binomial distributions. The generalized estimating equation technique was used to determine if amiodarone reduces the number of ventricular premature beats, the number of ventricular tachycardia episodes and the incidence of ventricular couplets compared to the control treatment. Results were presented as median, interquartile range (IQR) and proportion of reduction with 95% confidence intervals (CI) for continuous variables; for categorical variables, data were presented as proportion and relative risk with 95%CI. This meta-analysis was conducted in R statistical software version 3.3.2 [21], package geepack version 1.2–1 [22–24].

Random effects model meta-analysis of proportion, with logit transformation, using DerSimonian and Laird as variance estimator, was performed to determine side effects, and the results are presented as pooled prevalence, with 95%CI. This meta-analysis was conducted in R statistical software version 3.3.2, package meta version 4.8–1 [25].

## Results

### Description of studies

Nine studies [26–34] from 417 records found in the database search fulfilled eligibility criteria and were included in this systematic review, providing data from 365 subjects. There were three before-after studies [30,32,34], five case series (1 of which as follow period of a cluster randomized trial) [26,27,29,31,33] and one randomized clinical trial [28]. The mean age of participants ranged across the trials from 15 to 78 years. In 8 studies [26–33], amiodarone was administered orally and the dose ranged from 200 to 1200 mg, with a mean treatment time ranging from 1 day to 27 months. In 1 study [34], the dose of amiodarone was 5 mg/kg, given intravenously.

**Fig 1:**
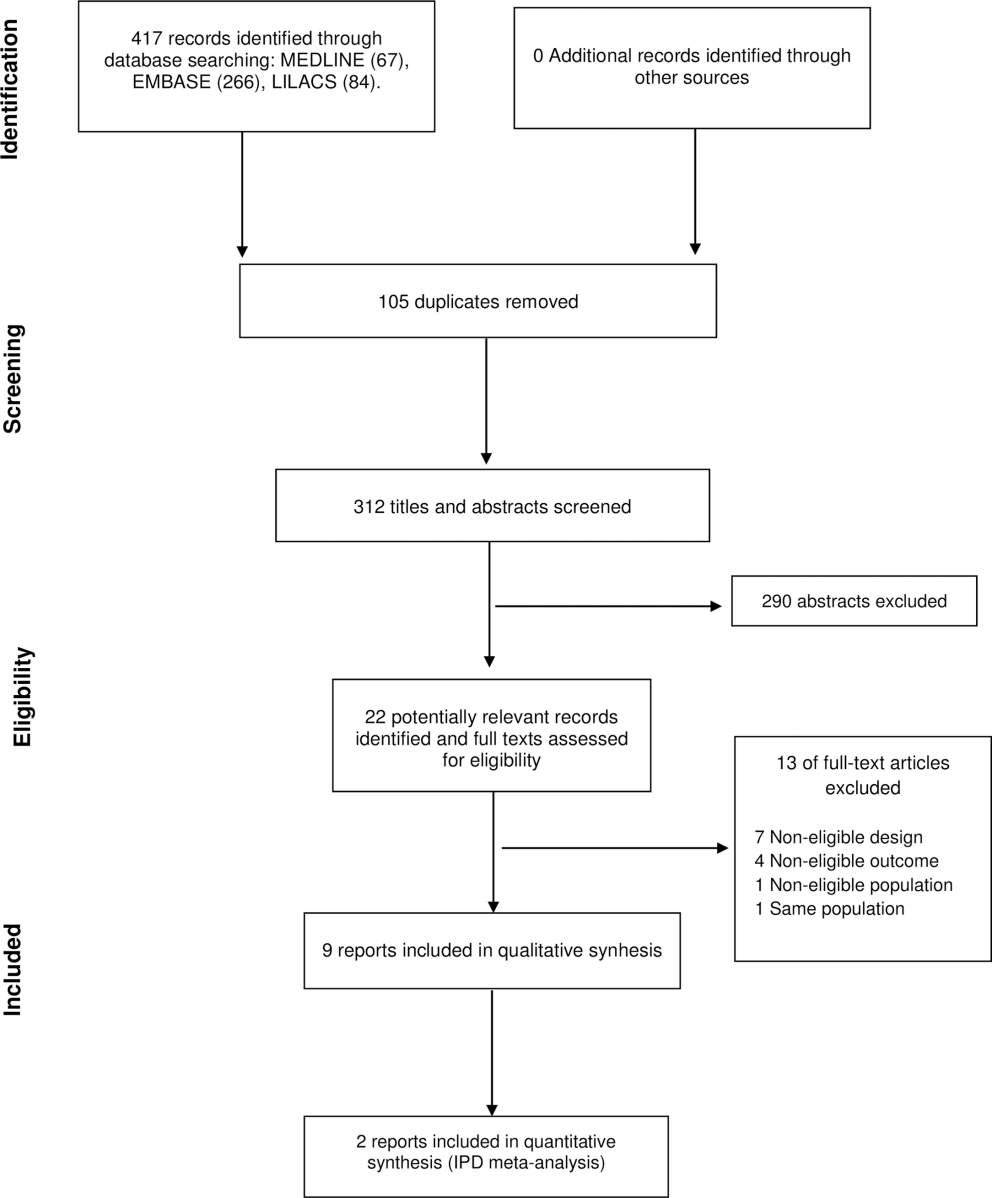
Flowchart of included studies.

Fig 1 shows the flow diagram of the selection process, and Table 1 summarizes the characteristics of the included studies.

**Table 1:** Characteristics of studies included in systematic review.

Author, Year, Country	Design	Population	N	Median age (Range)	Male	Intervention	Outcomes	Follow-up (months)
Belotti et al., 1983, Brazil [34]	Before-after study	Chagas patients with chronic cardiomyopathy	14	40.5 (28–77)	71%	Amiodarone, 900 to 1050 mg intravenous continuous infusion.	Ventricular tachycardia; Ventricular premature beats; Side effects Arrhythmias were measured by 24-hour Holter.	1 day
Chiale et al., 1984, Argentina [32]	Before-after study	Chagas patients with chronic myocarditis	24	51.5 (33–74)	-	Amiodarone, 600 to 800 mg/day in one or more daily doses, orally	Ventricular tachycardia; Ventricular premature beats; Ventricular couplets; Sudden death; Side effects Arrhythmias were measured by 24-hour Holter.	mean 26.6 (from 2 to 55)
Haedo et al., 1986, Argentina [30]	Before-after study	Chagas patients with chronic myocarditis	14	45.4 (30–61)	50%	Amiodarone, 800 mg/day in four doses	Ventricular tachycardia; Ventricular premature beats; Ventricular couplets; Side effects Arrhythmias were measured by 24-hour Holter.	1
Greco et al., 1980, Brazil [31]	Case series	G1: Chagas patients with frequent ventricular premature beats G2: Chagas patients with paroxysmal ventricular tachycardia	48 G1:34 G2:14	G1: 28.5 (22–68) G2: 37.1 (33–70)	G1: 42% G2: 18%	G1: amiodarone 600 mg/day G2: amiodarone 80 mg/day, and then 400 mg/day	Ventricular tachycardia; Ventricular premature beats; Sudden death; Side effects Arrhythmias were measured by conventional electrocardiogram.	from 4 to 9
Prata et al., 1982, Brazil [29]	Case series	Chagas patients with supraventricular and ventricular arrhythmias	120	(32–65)	40%	G1: amiodarone, 400 to 800 mg/day G2: amiodarone, 400 to 800 mg/day	Ventricular premature beats; Side effects. Arrhythmias were measured by conventional electrocardiogram.	2
Scanavacca et al., 1990, Brazil [27]	Case series	Chagas patients with chronic myocarditis and sustained ventricular tachycardia	35	50.0 (32–78)	69%	Amiodarone, during hospitalization, between 600 and 1200 mg/day	Ventricular tachycardia; Side effects; Sudden death Arrhythmias were measured by 24-hour Holter.	mean 27 (from 6 to 80)
Vichi et al., 1984, Brazil [26]	Case series	G1: Chagas patients with ventricular extra systoles G2: Chagas patients with significant ventricular extra systoles	20 G1:10 G2:10	G1: 43.5 (38–50) G2: 45,6 (39–51)	G1: 70% G2: 50%	G1: amiodarone 400 mg/day in 2 doses, orally G2: amiodarone 150 mg, intravenously for 1 minute, followed by 5 mg/kg/hour, intravenously for 120 minutes	Ventricular premature beats; Side effects. Arrhythmias were measured by conventional electrocardiogram.	1
Carrasco et al., 1985, Venezuela [33]	Case series (followed period of a cluster randomized trial)	Chagas patients with chronic myocarditis	9	51.0 (40–70)	67%	Amiodarone, 200 mg	Ventricular tachycardia; Ventricular premature beats; Side effects. Arrhythmias were measured by 24-hour Holter.	0.5
Rosenbaum et al., 1987, Argentina [28]	Randomized clinical trial (parallel design)	Chagas patients with ventricular arrhythmias	81 G1:40 G2:41	44.1 (15–65)	44%	G1: amiodarone, 800 mg/day G2: flecainide, 200 mg/day	Ventricular tachycardia; Ventricular premature beats; Ventricular couplets; Side effects. Arrhythmias were measured by 24-hour Holter and conventional electrocardiogram.	2

For data synthesis, only 2 studies [30,32] were included in the IPD meta-analysis for ventricular premature beats, ventricular tachycardia and ventricular couplets. Other studies [26–29,31,33,34] had different designs or presented the results in a non-comparable manner; therefore, these results were described narratively (Table 2).

**Table 2:** Narrative results.

Author, year	Effect on arrhythmia	Side effects
Belotti et al., 1983 [34]	**Ventricular premature beats**: 83.3% (10/12) of patients showed a reduction in the number of ventricular premature beats. **Ventricular tachycardia**: Ventricular tachycardia was suppressed in 33.3% (2/6) of patients.	28.6% (4/14) of patients had sinus bradycardia.
Chiale et al., 1984 [32]	**Ventricular premature beats**: 100% (24/24) of patients showed a reduction in the number of ventricular premature beats. **Ventricular tachycardia**: 100% (24/24) of patients had a reduction in the number of ventricular tachycardia episodes. **Ventricular couplets:** 91.7% (22/24) of patients had a reduction in the number of ventricular couplets. **Sudden death**: 4.17% (1/24) of patients experienced sudden death.	100% (24/24) of patients had corneal microdeposits, 8.33% (2/24) gastrointestinal events, 8.33% (2/24) sinus bradycardia, 8.33% (2/24) dermatological events, while 4.17% (1/24) discontinued treatment.
Haedo, et al., 1986 [30]	**Ventricular premature beats**: 100% (14/14) of patients had reduction in the number of ventricular premature beats. **Ventricular tachycardia**: 100% (14/14) of patients had a reduction in the number of ventricular tachycardia episodes. **Ventricular couplets:** 42.9% (6/14) of patients had a reduction in the number of ventricular couplets.	21.4% (3/14) of patients had corneal microdeposits, 28.6% (4/14) gastrointestinal events and 57.1% (8/14) sinus bradycardia.
Greco et al., 1980 [31]	**Ventricular premature beats**: 35.3% (12/34) of patients had a great response, 58.8% (20/34) a good response and 5.88% (2/34) a regular or no response*. **Ventricular tachycardia**: 85.7% (12/14) of patients had a great response*. **Sudden death**: 10.7% (5/48) of patients experienced sudden death.	100% (48/48) of patients had corneal microdeposits and 2.08% (1/48) sinus bradycardia, while 2.08% (1/24) of patients discontinued treatment.
Prata et al., 1982 [29]	**Ventricular premature beats**: 58.3% (70/120) of patients had a great response, 27.5% (33/120) a good response, 12.5% (15/120) a regular response and 1.70% (2/120) no response**.	Some patients had corneal microdeposits, gastrointestinal events, sinus bradycardia and dermatological events. Article did not report the number of patients with side effects.
Scanavacca et al., 1990 [27]	**Ventricular tachycardia:** The probability to suppress ventricular tachycardia was 62.0% in 12 months, 56.0% in 24 months and 44.0% in 36 months, with regular use of amiodarone. **Sudden death**: 5.71% (2/35) of patients experienced sudden death.	5.71% (2/35) of patients had corneal microdeposits, 2.86% (1/35) sinus bradycardia, 17.1% (6/35) dermatological events, 5.71% (2/35) pneumonitis and 2.86% (1/35) hypothyroidism, while 11.4% (4/35) of patients discontinued treatment.
Vichi et al., 1984 [26]	**Ventricular premature beats**: The proportion of premature beats decreased from 36.6% of total beats during the control phase to 6.10% after 4 weeks with oral amiodarone (10 patients) The proportion of premature beats decreased from 31.0% of total beats during the control phase to 4.24% after 120 minutes of intravenous amiodarone treatment (10 patients).	No undesirable side effects were observed.
Carrasco et al., 1985 [33]	**Ventricular premature beats**: Antiarrhythmic effect was total in 67% (6/9) of patients, partial in 11% (1/9) and insignificant in 22% (2/9). **Ventricular tachycardia:** Antiarrhythmic effect was total in 25% (2/9) of patients, partial in 62% (6/9) and ineffective in 13% (1/9).	11.1% (1/9) of patients had gastrointestinal events and 11.1% (1/9) sinus bradycardia, while 11.1% (1/9) of patients discontinued treatment.
Rosenbaum et al., 1987 [28]	**Ventricular premature beats**: Amiodarone: 90.7%(36/40) of patients had a reduction in the number of ventricular premature beats. Flecainide: 92.4%(38/41) of patients had a reduction in the number of ventricular premature beats. **Ventricular tachycardia:** Amiodarone: 92.6%(37/40) of patients had a reduction in the number of ventricular tachycardia. Flecainide: 96.5%(40/41) of patients had a reduction in the number of ventricular tachycardia episodes. **Ventricular couplets:** Amiodarone: 95.2%(38/40) of patients had a reduction in the number of ventricular couplets. Flecainide: 92.5%(38/41) of patients had a reduction in the number of ventricular couplets.	Amiodarone: 47.5% (19/40) of patients had corneal microdeposits, 5.00% (2/40) and dermatological events, while 7.50% (3/40) of patients discontinued treatment. Flecainide: 2.43% (1/41) of patients had sinus bradycardia, and 7.31% (3/41) of patients discontinued treatment.

* Great response defined as total disappearance of arrhythmias, good response as disappearance of 50 to 75% of arrhythmias, regular response as disappearance of 25 to 50% of arrhythmias and no response as arrhythmias down to 25%.

**Great response defined as total disappearance of arrhythmias, good response as disappearance of >50% of arrhythmias, regular response as disappearance of <50% of arrhythmias and no response as no change.

### Methodological quality

Among the before-after studies included in this systematic review, 62.5% were classified as fair quality and 37.5% as poor. All of them had a representative patient population, clear intervention description and outcome measures specified. However, they presented issues concerning lack of data about objective eligibility criteria, sample size, outcome assessor blinding, loss of follow-up and statistical analysis.

Regarding the randomized clinical trial, the study was conducted with a parallel design and showed high risk of bias, according to the RoB 2.0 tool. The study exhibited low risk for bias arising from intended interventions, measurement outcome data and selection of reported results, some concerns for randomization process and high risk of bias regarding measurement of the outcome. Complete assessment of risk of bias is presented in S3 Appendix.

### Effects of interventions

#### Ventricular tachycardia

Seven studies [27,28,30–34] evaluated ventricular tachycardia, and two studies [30,32] were included in the IPD meta-analysis. In these two studies, the median number of ventricular tachycardia episodes in 24-hour Holter was 25 (IQR 3–856) in the control group and 0 (IQR 0–0) for the intervention group (Table 3). Amiodarone reduced ventricular tachycardia in 99.9% (95%CI 99.8–100% in 24-hour Holter monitoring) (Table 3). All studies that were not included in the meta-analysis reported a decrease in the number of ventriculartachycardia episodes after the use of amiodarone (Table 2). The quality of evidence according to GRADE for this outcome was moderate. (S4 Appendix).

**Table 3:** Ventricular tachycardia, ventricular premature beats and ventricular couplets after amiodarone.

**Ventricular tachycardia (no./24 hours)**	**Control (Median (IQR))**	**Amiodarone (Median (IQR))**
Chiale et al., 1984 [32]	233 (2.8–481.5)	0 (0–0)
Haedo et al., 1986 [30]	12 (3.5–512.5)	0 (0–0)
Median (IQR) *raw*	25 (3.00–856)	0 (0–0) *
Ventricular tachycardia reduction (95%CI) *adjusted*	**—**	99.9% (95%CI 99.8–100%)
**Ventricular premature beats (no./24 hours)**	**Control (Median (IQR))**	**Amiodarone (Median (IQR))**
Chiale et al., 1984 [32]	10.4 (8.01–26.5)	7115.5 (25–348.2)
Haedo et al., 1986 [30]	6.12 (5.70–17.7)	321 (103.5–887.2)
Median (IQR) *raw*	8924.5 (5987.5–21295.5)	161.5 (34.3–560.3)
Ventricular premature beats reduction (95%CI) *adjusted*	**—-**	93.1% (95%CI 82–97.4%)
**Incidence of ventricular couplets (24h)**	**Control (N)**	**Amiodarone (N)**
Chiale et al., 1984 [32]	24/24 (100%)	2/24 (8.3%)
Haedo et al., 1986 [30]	14/14 (100%)	6/14(42.9%)
Relative risk (95%CI) *adjusted*	1	0.21 (0.11–0.39)

* Out of 38 patients, only 1 showed ventricular tachycardia (a single episode) during 24-hour Holter, after use of amiodarone.

#### Ventricular premature beats

Eight studies [26,28–34] evaluated ventricular premature beats and two studies [30,32] were included in the IPD meta-analysis. In these two studies, the median number of ventricular premature beats in 24-hour Holter was 8924.5 (IQR 5987.5–21295.5) in the control group and 161.5 (IQR 34.3–560.3) for the intervention group (Table 3). Amiodarone reduced ventricular premature beats in 93.1% (95%CI 82–97.4% in 24-hour Holter monitoring) (Table 3). All studies that were not included in the meta-analysis reported a decrease in the number of ventricular premature beats after the use of amiodarone (Table 2). The quality of evidence according to GRADE for this outcome was moderate (S4 appendix).

#### Ventricular couplets

Three studies [28,30,32] assessed ventricular couplets and two of them [30,32] were included in the IPD meta-analysis. In the control group, 38 out of 38 patients had ventricular couplets (100%), while 8 out of 38 patients (21.0%) had it in the intervention group (Table 3). Amiodarone reduced the incidence of ventricular couplets by 79.0% (RR 0.21, 95%CI 0.11–0.39 in 24-hour Holter monitoring) (Table 3). In the study that was not included in the meta-analysis, 95.2% (38/40) of patients showed a reduction in the number of ventricular couplets with use of amiodarone [28]. The quality of evidence according to GRADE for this outcome was moderate (S4 appendix).

#### Sudden death

Three studies [27,31,32] (n = 107) evaluated sudden death after the use of amiodarone. Eight patients (7.50%) experienced sudden death after 5 to 27 months of treatment. The quality of evidence according to GRADE for this outcome was very low (S4 appendix).

#### Side effects

All studies [26–34] investigated side effects caused by amiodarone and 7 were included in the meta-analysis [27,28,30–34]. The studies not included in the meta-analysis are described in Table 2. Five studies [27,28,30–32] assessed corneal microdeposits, three studies [30–32] reported cases of gastrointestinal events, six studies [27,30–34] reported cases of sinus bradycardia, three studies assessed dermatological events [27,28,32] and five studies assessed drug discontinuation [27,28,31–33].

The prevalence of corneal microdeposits after use of amiodarone was 61.0% (95%CI 18.8–91.3%, n = 161), gastrointestinal events 16.1% (95%CI 6.61–34.2%, n = 47), sinus bradycardia 12.6% (95%CI 3.68–35.4%, n = 144), dermatological events 10.5% (95%CI 4.87–21.3%, n = 99), and drug discontinuation 7.59% (95%CI 4.12–13.6%, n = 156) (S5 Appendix). Three studies assessed hypo- or hyperthyroidism (n = 203), where only one patient showed hypothyroidism [27,29,31]. One study reported two cases of pneumonitis during the use of amiodarone [27].

## Discussion

This systematic review included 9 studies that determined the effects of amiodarone with regard to arrhythmia, side effects and sudden death in individuals with Chagas disease. Due to the high heterogeneity on outcome assessment and diversity of study designs, most results were presented qualitatively. However, it was possible to pool two before-after studies in an IPD meta-analysis, since these studies provided the full dataset in the published articles [30,32]. For adverse side effects, available data allowed pooling frequency estimates using the conventional meta-analysis approach for seven studies [27,28,30–34].

According to our findings, there was moderate certainty in the body of evidence for the effect of amiodarone on the clinically significant reduction of arrhythmia in patients with Chagas disease, such as ventricular tachycardia episodes (reduction of 99%), ventricular premature beats (reduction of 93%) and ventricular couplets (RR = 0.21). Although it was only possible to pool two studies in the IPD metanalysis, all other studies showed similar effects on these outcomes.

Despite the strong evidence for arrhythmia reduction, studies did not provide enough information for the assessment of the effect on clinically relevant outcomes, such as mortality and hospitalization. Arrhythmia was mainly assessed using 24-hour Holter or conventional electrocardiogram evaluation, and it was expected that most episodes would be asymptomatic, without any clinical impact. Although clinical benefit is expected with arrhythmia reduction, this information should be interpreted with caution, since arrhythmia is a surrogate outcome, and is not clear the clinical impact on death and hospitalization reduction over time. Little information was identified related to hard endpoints; three studies, with 107 patients, observed 8 sudden deaths (7.50%) after 5 to 27 months of treatment, without comparison with any control group.

Effects of amiodarone on sudden death are available from different clinical conditions. Piccini et al. performed a meta-analysis of studies using amiodarone for primary prophylaxis of sudden cardiac death in high-risk patients (post myocardial infarction, heart failure). Compared to placebo/control, there was a 29 and 18% reduction in sudden death and cardiovascular mortality, respectively [35]. Moreover, in a recent meta-analysis of primary prevention, amiodarone reduced the risk of sudden cardiac death by 34% [14]. In the absence of direct evidence for patients with Chagas cardiomyopathy, these figures may provide an indirect estimate of the potential benefit in our population of interest.

Regarding side effects, our systematic review observed that amiodarone was associated with corneal microdeposits, gastrointestinal events, sinus bradycardia, dermatological events, pneumonitis, hypothyroidism and drug discontinuation. Other studies that did not include Chagas patients suggested that adverse effects are common, with a prevalence as high as 15% in the first year of use and 50% during long-term use, indicating a cumulative effect with chronic use, corroborating our results [36]. Moreover, in an additional study, the risk of discontinuation of amiodarone was 45% superior than placebo, however presenting similar rates when compared with other antiarrhythmics [14]. Of note, even though our review found that amiodarone was associated with a low incidence of sinus bradycardia (7%), concomitant use of amiodarone and beta-blockers in Chagas cardiomyopathy may increase the risk of exacerbation of bradyarrhythmia [37].

There are other interventions with potential benefit for patients with Chagas disease and arrhythmias. Observational studies have shown that an implantable cardioverter defibrillator is effective for primary or secondary prevention of death in Chagas disease [38,39]. However, there are obstacles concerning its use due to socioeconomic limitations such as low income and poor health and nutrition conditions. The first randomized clinical trial comparing the implantable cardioverter defibrillator and amiodarone for primary prevention of death in patients with Chagas disease and nonsustained ventricular tachycardia, the CHAGASICS study, is ongoing and may provide important information for the field in the near future [40].

Our systematic review and meta-analysis has several strengths. We performed IPD meta-analysis, which has potential advantages such as increased power and reduced bias, and it is considered the gold standard in evidence synthesis [41]. Moreover, our comprehensive search of various databases, along with seeking the information from study authors and experts in the field, makes it unlikely that any relevant study was missed.

However, our review has some limitations, especially related to the nature of the available data. Most of our results are based on limited observational data. All included studies were small and presented methodological limitations, and the majority of them presented a short-term follow-up.

Another fact that may reduce the external validity of our findings is that all included studies were published from 1980 to 1990. In the last 30 years, the treatment of heart failure and arrhythmias has progressed exponentially. The addition of ACE inhibitors, beta-blockers and mineralocorticoid antagonists has modified the evolution of heart failure, and the emergence of electrophysiology as well has added the use of ablation therapies and implantable cardio-defibrillators in the management of patients with Chagas cardiomyopathy. There is a gap in the literature showing the effect of such developments on the number of events or mortality in these patients.

An important aspect to highlight is the lack of interest of the scientific community regarding this topic, where the most recent article included in the review was published in 1990, and the absence of systematic reviews targeting this research question. Our study demonstrates the lack of clinical trials and observational data on therapeutic options for chagasic cardiomyopathy considering the current therapeutic arsenal.

In spite of data limitations, the estimates reported here show that amiodarone seems to be an effective antiarrhythmic drug for patients with Chagas disease, reducing uncomfortable symptoms such as palpitations and potentially fatal events related to ventricular tachycardia. However, it is important to stress that there is no direct evidence for the reduction on clinically relevant outcomes. Our findings may be useful to support clinicians’ decision-making in amiodarone use, especially in settings where an implantable cardioverter defibrillator is not available or affordable, but there is a need to balance the potential benefits and harms at the individual level.

## Supporting information

S1 Checklist PRISMA checklist. (PDF)

S1 Appendix PROSPERO protocol. (PDF)

S2 Appendix Search strategy. (PDF)

S3 Appendix Risk of bias assessment. (PDF)

S4 Appendix GRADE assessment. (PDF)

S5 Appendix Side effects. (PDF)

## References

[1] Bern C. Chagas' Disease. The New England Journal of Medicine. 373 2015;373(5):456–466. 10.1056/NEJMra1410150 26222561

[2] Henao-Martinez AF, Colborn K, Parra-Henao G. Overcoming research barriers in Chagas disease-designing effective implementation science. Parasitol Res. 2017;116: 35–44. 10.1007/s00436-016-5291-z 27771804

[3] Jackson Y, Pinto A, Pett S. Chagas disease in Australia and New Zealand: risks and needs for public health interventions. Trop Med Int Health. 2014;19: 212–218. 10.1111/tmi.12235 24299280

[4] Gascon J, Bern C, Pinazo MJ. Chagas disease in Spain, the United States and other non-endemic countries. Acta Trop. 2010;115: 22–27. 10.1016/j.actatropica.2009.07.019 19646412

[5] World Health Organization. What is Chagas disease? 2018 In: World Health Organization [Internet]. Available from: http://www.who.int/chagas/disease/en/.

[6] Chatelain E. Chagas disease research and development: Is there light at the end of the tunnel? Comput Struct Biotechnol J. 2017;15: 98–103. 10.1016/j.csbj.2016.12.002 28066534 PMC5196238

[7] Ribeiro AL, Nunes MP, Teixeira MM, Rocha MO. Diagnosis and management of Chagas disease and cardiomyopathy. Nat Rev Cardiol. 2012;9: 576–589. 10.1038/nrcardio.2012.109 22847166

[8] Yancy CW, Jessup M, Bozkurt B, Butler J, Casey DE Jr., et al 2013 ACCF/AHA guideline for the management of heart failure: a report of the American College of Cardiology Foundation/American Heart Association Task Force on Practice Guidelines. J Am Coll Cardiol. 2013;62:e147–239. 10.1016/j.jacc.2013.05.019 23747642

[9] Morillo CA, Marin-Neto JA, Avezum A, Sosa-Estani S, Rassi A Jr., et al Randomized Trial of Benznidazole for Chronic Chagas' Cardiomyopathy. N Engl J Med. 2015;373: 1295–1306. 10.1056/NEJMoa1507574 26323937

[10] Bestetti RB, Cardinalli-Neto A. Device therapy in Chagas disease heart failure. Expert Rev Cardiovasc Ther. 2012;10: 1307–1317. 10.1586/erc.12.115 23190069

[11] Rassi Junior A, Gabriel Rassi A, Gabriel Rassi S, Rassi Junior L, Rassi A. Ventricular arrhythmia in Chagas disease. Diagnostic, prognostic, and therapeutic features. Arq Bras Cardiol. 1995;65: 377–387. 8728815

[12] Rassi A Jr., Rassi SG, Rassi A. Sudden death in Chagas' disease. Arq Bras Cardiol. 2001;76: 75–96. 11175486 10.1590/s0066-782x2001000100008

[13] Scanavacca MI, de Brito FS, Maia I, Hachul D, Gizzi J, et al Guidelines for the evaluation and treatment of patients with cardiac arrhythmias. Arq Bras Cardiol. 2002; 79 Suppl 5: 1–50. 12700835

[14] Claro JC, Candia R, Rada G, Baraona F, Larrondo F, et al (2015) Amiodarone versus other pharmacological interventions for prevention of sudden cardiac death. Cochrane Database Syst Rev. 12:CD008093. 10.1002/14651858.CD008093.pub2 PMC8407095 26646017

[15] Benaim G, Sanders JM, Garcia-Marchan Y, Colina C, Lira R, et al Amiodarone has intrinsic anti-Trypanosoma cruzi activity and acts synergistically with posaconazole. J Med Chem. 2006;49: 892–899. 10.1021/jm050691f 16451055

[16] Moher D, Liberati A, Tetzlaff J, Altman DG, Group P. Preferred reporting items for systematic reviews and meta-analyses: the PRISMA Statement. Open Med. 2009;3: e123–130. 21603045 PMC3090117

[17] Higgins JPT, Green S. Cochrane Handbook for Systematic Reviews of Interventions, version 5.1.0. The Cochrane Collaboration, 2011. Available from: 409 http://handbook.cochrane.org.

[18] Higgins JPT, Sterne JAC, Savović J, Page MJ, Hróbjartsson A, Boutron I, et al A revised tool for assessing risk of bias in randomized trials. 2016;10(Suppl1).

[19] NIH. Quality Assessment Tool for Before-After (Pre-Post) Studies With No 413 Control Group. 2014. Available from: https://www.nhlbi.nih.gov/health-414topics/study-quality-assessment-tools.

[20] Iorio A, Spencer FA, Falavigna M, Alba C, Lang E, et al Use of GRADE for assessment of evidence about prognosis: rating confidence in estimates of event rates in broad categories of patients. BMJ. 2015;350: h870 10.1136/bmj.h870 25775931

[21] R Development Core Team. R: a language and environment for statistical computing R Foundation for Statistical Computing 2008

[22] Yan J, Fine J. Estimating equations for association structures. Stat Med. 2004;23: 859–874. 10.1002/sim.1650 15027075

[23] Yan J. Yet Another Package for Generalized Estimating Equations. R-News. 2002;2: 12–14.

[24] Halekoh U, Højsgaard S, J. Y. The R Package geepack for Generalized Estimating Equations. Journal of Statistical Software, 2006;15: 1–11.

[25] Schwarzer G. Meta: An R package for meta-analysis. R News. 2007

[26] Vichi FL. Anti-arrhythmia effect of oral and injectable amiodarone in ventricular extrasystole of chronic Chagas’ disease patients. RBM cardiol. 1984;3: 40–44.

[27] Scanavacca MI, Sosa EA, Lee JH, Bellotti G, Pileggi F. Empiric therapy with amiodarone in patients with chronic chagas cardiomyopathy and sustained ventricular tachycardia. Arq Bras Cardiol. 1990;54: 367–371. 2288524

[28] Rosenbaum M, Posse R, Sgammini H, Núnez Burgos J, Chiale PA, et al Flecainide and amiodarone in the treatment of ventricular arrhythmias associated with chagas heart disease. Arch Inst Cardiol Mex. 1987;57: 325–330. 2445316

[29] Prata SP, Batista EP, Penhalver JR. Use of amiodarona in cardiac arrhytimias caused by chronic Chagas’ disease. Folha Med. 1982;85: 713–717.

[30] Haedo AH, Chiale PA, Bandieri JD, Lazzari JO, Elizari MV, et al Comparative antiarrhythmic efficacy of verapamil, 17-monochloracetylajmaline, mexiletine and amiodarone in patients with severe chagasic myocarditis: relation with the underlying arrhythmogenic mechanisms. J Am Coll Cardiol, 1986;7: 1114–1120. 3958370 10.1016/s0735-1097(86)80232-7

[31] Greco OT, Lorga AM, Garzon SA, Younan I, Bellini AJ, et al Amiodarone in ventricular arrhythmias of chronic Chagas cardiopathy. Arq Bras Cardiol. 1980;35: 177–181. 7213094

[32] Chiale PA, Halpern MS, Nau GJ, Tambussi AM, Przybylski J, et al Efficacy of amiodarone during long-term treatment of malignant ventricular arrhythmias in patients with chronic chagasic myocarditis. Am Heart J. 1984;107: 656–665. 6702559 10.1016/0002-8703(84)90311-9

[33] Carrasco HA, Vicuna AV, Molina C, Landaeta A, Reynosa J, et al Effect of low oral doses of disopyramide and amiodarone on ventricular and atrial arrhythmias of chagasic patients with advanced myocardial damage. Int J Cardiol. 1985;9: 425–438. 3908329 10.1016/0167-5273(85)90238-4

[34] Bellotti G, Silva LA, Esteves Filho A, Gruppi C, Rati M, et al Electrocardiographic and hemodynamic effects of amiodarone hydrochloride by intravenous route. Arq Bras Cardiol. 1983;40: 141–144. 6354147

[35] Piccini JP, Berger JS, O'Connor CM. Amiodarone for the prevention of sudden cardiac death: a meta-analysis of randomized controlled trials. Eur Heart J. 2009; 30: 1245–1253. 10.1093/eurheartj/ehp100 19336434

[36] Raeder EA, Podrid PJ, Lown B. Side effects and complications of amiodarone therapy. Am Heart J. 1985;109: 975–983. 3158188 10.1016/0002-8703(85)90238-8

[37] Gali WL, Sarabanda AV, Baggio JM, Ferreira LG, Gomes GG, et al Implantable cardioverter-defibrillators for treatment of sustained ventricular arrhythmias in patients with Chagas' heart disease: comparison with a control group treated with amiodarone alone. Europace. 2014;16: 674–680. 10.1093/europace/eut422 24481778

[38] Martinelli M, de Siqueira SF, Sternick EB, Rassi A Jr., Costa R, et al Long-term follow-up of implantable cardioverter-defibrillator for secondary prevention in chagas' heart disease. Am J Cardiol. 2012;110: 1040–1045. 10.1016/j.amjcard.2012.05.040 22727179

[39] Cardinalli-Neto A, Nakazone MA, Grassi LV, Tavares BG, Bestetti RB. Implantable cardioverter-defibrillator therapy for primary prevention of sudden cardiac death in patients with severe Chagas cardiomyopathy. Int J Cardiol. 2011;150: 94–95. 10.1016/j.ijcard.2011.03.036 21497920

[40] Martinelli M, Rassi A Jr., Marin-Neto JA, de Paola AA, Berwanger O, et al CHronic use of Amiodarone aGAinSt Implantable cardioverter-defibrillator therapy for primary prevention of death in patients with Chagas cardiomyopathy Study: rationale and design of a randomized clinical trial. Am Heart J. 2013;166: 976–982. 10.1016/j.ahj.2013.08.027 24268211

[41] Debray TP, Moons KG, van Valkenhoef G, Efthimiou O, Hummel N, et al Get real in individual participant data (IPD) meta-analysis: a review of the methodology. Res Synth Methods. 2015;6: 293–309. 10.1002/jrsm.1160 26287812 PMC5042043

