# Supplementary material for: Amiodarone for arrhythmia in patients with Chagas disease: A systematic review and individual patient data meta-analysis
Source: PLoS Negl Trop Dis. 2018 Aug 20;12(8):e0006742. doi: 10.1371/journal.pntd.0006742 (PMC6130878; doi:10.1371/journal.pntd.0006742)
Supplement: S1 Appendix — (PDF) [file pntd.0006742.s002.pdf]

Amiodarone for arrhythmia in patients with cardiac form of Chagas disease: a systematic review

Cinara Stein, Celina Migliavaca, Veronica Colpani, Luciane Cruz, Maicon Falavigna

Citation

Cinara Stein, Celina Migliavaca, Veronica Colpani, Luciane Cruz, Maicon Falavigna. Amiodarone for arrhythmia in patients with cardiac form of Chagas disease: a systematic review. PROSPERO 2017 CRD42017056765 Available from:
http://www.crd.york.ac.uk/PROSPERO/display_record.php?ID=CRD42017056765

Review question

The primary objective of this systematic review is to evaluate the effect of amiodarone in arrhythmia in patients with cardiac form of chronic Chagas disease.

Searches

We will search MEDLINE (accessed by PubMed), EMBASE and LILACS databases to retrieve potentially relevant articles.
Moreover, we will search on the website ‘clinicaltrials.gov’ and scan the reference lists of identified publications for additional studies.
Search terms will include relevant headings and keywords in the title, abstract and text, including terms as “Chagas’ disease”, “Trypanosoma cruzi” and “amiodarone”.
Keywords related to outcomes of interest and publication type will not be included to enhance sensitivity of search.

Types of study to be included

Observational investigations (cohort studies or case series) and intervention studies (quasi-experiments and randomized clinical trials) will be included.We will exclude experimental studies, case-control studies, cross-sectional studies, systematic reviews and meta-analyses, letters, and editorials.

Condition or domain being studied

Considered a neglected tropical disease, Chagas disease is caused by the Trypanosoma cruzi (T. cruzi), a protozoan parasite. Chagas disease affects about 6 million people in 21 countries of Latin American, and has recently become a global health concern (1), especially due to immigration from endemic areas into the developed world (2). In Latin America, Chagas heart disease is still a major cause of heart failure despite a drop-in incidence in the last decades (4)
During the chronic phase of the disease, some patients develop cardiac complications, which can be manifest as heart failure, segmental wall motion abnormalities (aneurysms), thrombo-embolic events, conduction system disturbances, ventricular arrhythmias (VAs), and sudden death (5,6).
Cardiac arrhythmias are common in patients with Chagas cardiomyopathy and amiodarone has been widely used as antiarrhythmic drug. Amiodarone has been recommended as the treatment of choice for all patients with sustained ventricular tachycardia, and also for those with no sustained ventricular tachycardia with myocardial dysfunction (6); however, frequent occurrence of adverse effects is an important concern when deciding on its use (7). Moreover, only few studies analyzed the effects of amiodarone in the treatment of arrhythmia in patients with Chagas disease (8). Based on this, it is necessary to systematically review the effects of amiodarone in arrhythmia of chagasic patients.
1. World Health Organization. Research priorities for Chagas disease, human African trypanosomiasis and leishmaniasis. World Health Organ Tech Rep Ser 2012: v–xii, 1–100. 2.
2. Bern C, Montgomery SP, Herwaldt BL, Rassi A, Marin-Neto JA, Dantas RO, Maguire JH, Acquatella H, Morillo C, Kirchhoff LV, Gilman RH, Reyes PA, Salvatella R, Moore AC. Evaluation and treatment of chagas disease in the United States: a systematic review. JAMA. 2007; 298:2171–81.

3. Chagas disease in Latin America: an epidemiological update based on 2010 estimates. Wkly Epidemiol Rec. 2015; 90:33-43.
4. Bocchi EA. Heart failure in South America. Curr Cardiol Rev. 2013; 9:147–56.
5. Marin Neto JA, Simões MV, Sarabanda AV. Chagas' heart disease. Arq Bras Cardiol 1999; 72:247–80.
6. Rassi A Jr, Rassi A, Marin-Neto JA. Chagas disease. Lancet 2010; 375:1388–402.
7. Park HS, Kim YN. Adverse effects of long-term amiodarone therapy. Korean J Inter Med. 2004; 29: 571-3.
8. Benaim G, Paniz Mandolfi AE. The emerging role of amiodarone and dronedarone in Chagas disease. Nat Rev Cardiol 2012; 9:605-9.

Participants/population
Adults (> 18 years old) affected by the cardiac chronic form of Chagas disease.

Intervention(s), exposure(s)
We will include studies assessing the use of amiodarone to treat arrhythmia in patients affected by Chagas disease.

Comparator(s)/control
The comparator groups will be placebo group, standard care, no intervention, or different treatment drugs.

Context
Studies that have included adults, of any gender, race or ethnic background, with a diagnosis of the cardiac form of Chagas disease and having clinical indications supporting the use of antiarrythmic drugs.

Primary outcome(s)
Arrhythmia, associated to Chagasic cardiac disease.

Secondary outcome(s)
Mortality, sudden death and side effects.

Data extraction (selection and coding)
Two reviewers (CS, CBM) will separately and independently screen the titles and abstracts of studies identified from initial searches. A standard screening checklist based on the eligibility criteria above will be used for each study. Studies that do not meet the criteria according to the titles or abstracts will be excluded. Full text versions of the remaining studies, including those that are potentially eligible studies and uncertain, will be retrieved for a second review, by at least two reviewers independently, to determine the eligibility. Disagreements with regard to study eligibility will be further discussed among reviewers. If consensus cannot be reached, a third reviewer (VC) will make the ultimate decision. For studies with insufficient data to evaluate the eligibility, we will contact the study authors by email for clarification. The studies will be excluded if there is still insufficient data after this contact. If more than one publication reports results from the same study population, we will choose the publication with the largest sample size or provides more information. Abstracts published in academic conferences will be evaluated case by case, and we will contact the study authors for details if necessary. The following data will be collected from each study: general study characteristics (title and authors, year of study, geographical location), methods (study design, participant allocation, measured outcomes reported, covariables), participant's characteristics (age, sex, study inclusion and exclusion criteria), intervention details (dose, period of use) and outcomes (primary and secondary outcomes, time follow-up, number of events). If the study is reported in duplicate, the study published earlier or which provides more information will be included. The same reviewers (CS, CBM) will separately and independently extract data from the eligible studies. Disagreements regarding data extraction between the authors will be resolved by discussion. If consensus cannot be reached, a third author (VC) will review the study and arbitrate. If data are missing for synthesis or assessment of study quality, we will attempt to contact the study authors by email at least twice. The study will be excluded if there is still insufficient data following this process.

Risk of bias (quality) assessment

Two review authors (CS, CBM) will independently assess for quality. Clinical trials and crossover studies will be evaluated according to RoB 2.0: a revised tool for risk of bias in randomized trials (Higgins JPT et al. Revised Cochrane risk of bias tool for randomized trials (RoB 2.0). Seoul Colloquium in October 2016). Observational studies will be assessed for quality using the 'Quality Assessment Tool for Before–After Studies with No Control Group' (NHLBI, RTI International. Quality Assessment Tool for Before-After (Pre-Post) Studies With No Control Group. 2014; https://www.nhlbi.nih.gov/health-pro/guidelines/in-develop/cardiovascular-risk reduction/tools/before-after; Accessed February, 2017). A table with details of risk of bias in individual studies will be provided.

Strategy for data synthesis

The results will be presented descriptively and the evidence will be incorporated into tabular displays. Variables will be synthesized narratively and summarized using descriptive statistics (frequencies, percentages).
If possible, we will perform a meta-analysis, using R program (version 3.2.3) and the meta and metafor package. Relative risk will be computed for the outcomes, and both fixed effects and random effects models will be applied, using the model appropriate to the determined methodological heterogeneity between studies. Heterogeneity between studies will be assessed using the Q statistic and the I-squared test. Publication bias across studies will be evaluated using funnel plots and Egger's test, and the overall quality of evidence will be assessed using GRADE.

Analysis of subgroups or subsets

If a sufficient number of trials are identified, a subgroup analysis will be performed according to the type of comparator.

Contact details for further information

Cinara Stein
cinarastein@gmail.com

Organisational affiliation of the review

Hospital Moinhos de Vento
http://www.hospitalmoinhos.org.br

Review team members and their organisational affiliations

Miss Cinara Stein. Hospital Moinhos de Vento
Miss Celina Migliavaca. Hospital Moinhos de Vento
Dr Veronica Colpani. Hospital Moinhos de Vento
Dr Luciane Cruz. Hospital Moinhos de Vento
Dr Maicon Falavigna. Hospital Moinhos de Vento

Collaborators

Miss Andreia Diel. Hospital Moinhos de Vento

Anticipated or actual start date

06 February 2017

Anticipated completion date

30 April 2017

Funding sources/sponsors

Programa de Apoio ao Desenvolvimento Institucional do Sistema único de Saúde (PROADI-SUS). Brazilian Ministry of Health.

Conflicts of interest

None known

Language
English

Country
Brazil

Stage of review
Review_Ongoing

Subject index terms status
Subject indexing assigned by CRD

Subject index terms
Amiodarone; Arrhythmias, Cardiac; Chagas Cardiomyopathy; Chagas Disease; Heart; Humans; Treatment Outcome

Date of registration in PROSPERO
06 February 2017

Date of publication of this version
06 February 2017

Details of any existing review of the same topic by the same authors

Stage of review at time of this submission

Stage	Started	Completed
Preliminary searches	Yes	Yes
Piloting of the study selection process	No	No
Formal screening of search results against eligibility criteria	No	No
Data extraction	No	No
Risk of bias (quality) assessment	No	No
Data analysis	No	No

Versions
06 February 2017

PROSPERO
This information has been provided by the named contact for this review. CRD has accepted this information in good faith and registered the review in PROSPERO. CRD bears no responsibility or liability for the content of this registration record, any associated files or external websites.
