# Supplementary material for: Amiodarone for arrhythmia in patients with Chagas disease: A systematic review and individual patient data meta-analysis
Source: PLoS Negl Trop Dis. 2018 Aug 20;12(8):e0006742. doi: 10.1371/journal.pntd.0006742 (PMC6130878; doi:10.1371/journal.pntd.0006742)
Supplement: S2 Appendix — (PDF) [file pntd.0006742.s003.pdf]

Search Strategy

Pubmed

1. "Chagas Disease"[Mesh]
2. "chagas disease”
3. "Trypanosoma cruzi"[Mesh]
4. "Trypanosoma cruzi"
5. 1 OR 2 OR 3 OR 4
6. " Amiodarone"[Mesh]
7. Amiodarone
8. 6 OR 7
9. 5 AND 8

Embase

1. 'chagas disease'/exp
2. 'chagas disease'
3. 'trypanosoma cruzi'/exp
4. 'trypanosoma cruzi'
5. 1 OR 2 OR 3 OR 4
6. ‘amiodarone’/exp
7. ‘amiodarone’
8. 6 OR 7
9. 5 AND 8

Lilacs

1. Chagas disease AND amiodarone
