# Supplementary material for: Amiodarone for arrhythmia in patients with Chagas disease: A systematic review and individual patient data meta-analysis
Source: PLoS Negl Trop Dis. 2018 Aug 20;12(8):e0006742. doi: 10.1371/journal.pntd.0006742 (PMC6130878; doi:10.1371/journal.pntd.0006742)
Supplement: S3 Appendix — (PDF) [file pntd.0006742.s004.pdf]

Methodological quality of the included studies using the 'Quality Assessment Tool for Before–After Studies with No Control Group'

Before -After studies													
Studies	1	2	3	4	5	6	7	8	9	10	11	12	*Quality rating*
Belotti et al., 1983	Y	N	Y	NR	NR	Y	Y	NR	NR	Y	NA	NA	Fair
Carrasco et al., 1985	Y	N	Y	NR	NR	Y	Y	NR	NR	Y	NA	NA	Fair
Chiale et al., 1984	Y	N	Y	NR	NR	Y	Y	NR	NR	Y	NA	NA	Fair
Greco et al., 1980	Y	N	Y	NR	NR	Y	Y	NR	NR	N	NA	NA	Poor
Haedo et al., 1986	Y	N	Y	NR	NR	Y	Y	NR	NR	Y	NA	NA	Fair
Prata et al., 1982	N	N	Y	NR	NR	Y	Y	NR	NR	N	NA	NA	Poor
Scanavaca et al., 1990	Y	N	Y	NR	NR	Y	Y	NR	NR	Y	NA	NA	Fair
Vichi et al., 1984	N	N	Y	NR	NR	Y	Y	NR	NR	N	NA	NA	Poor

Abbreviations: Y, yes; N, no; NA, not applicable; NR, not reported. (1) objective clearly stated; (2) eligibility criteria described; (3) representative patient population; (4) all eligible participants enrolled in study; (5) sample size sufficient; (6) intervention description; (7) outcome measures specified; (8) outcome assessor blinded; (9) loss to follow-up; (10) statistical analysis of outcome measures before and after intervention; (11) interrupted time series design; (12) individual data used for group-level effects.

Methodological quality of the included studies using RoB 2.0 tool (individually randomized, cross-over trials) and RoB 2.0 tool (individually randomized, parallel group trials)

**Study, year**	**Design**	**Randomization**	**Interventions**	**Missing outcome data**	**Outcome Measurement**	**Reported results**	**Overall**
Rosenbaum et al., 1987	Randomized Clinical trial (parallel design)	Some concerns	Low risk	Low risk	High risk	Low risk	High risk
