# Supplementary material for: Amiodarone for arrhythmia in patients with Chagas disease: A systematic review and individual patient data meta-analysis
Source: PLoS Negl Trop Dis. 2018 Aug 20;12(8):e0006742. doi: 10.1371/journal.pntd.0006742 (PMC6130878; doi:10.1371/journal.pntd.0006742)
Supplement: S4 Appendix — (PDF) [file pntd.0006742.s005.pdf]

Certainty assessment							Summary of findings
**No. of participants (studies) Follow-up**	**Risk of bias**	**Inconsistency**	**Indirectness**	**Imprecision**	**Others**	**Overall certainty of evidence**	
**Ventricular tachycardia**							
225 (6 observational studies and 1 randomized clinical trial)	serious [a]	not serious	not serious	not serious	very strong association	⨁⨁⨁◯ MODERATE	In IPD meta-analysis, amiodarone reduced ventricular tachycardia in 99.9% (95%CI 99.8-100% in 24-hour Holter monitoring.<br>Belloti et al., 1983 reported that antiarrhythmic effect was total in 33.33% (2/6).<br>Greco et al., 1980 reported that antiarrhythmic action was total in 85.71% (12/14).<br>Scanavacca et al., 1990 reported that the probability of suppressing ventricular tachycardia was 62% in 12 months, 56% in 24 months and 44% in 36 months, with regular use of amiodarone.<br>Carrasco et al., 1985 reported that antiarrhythmic effect was total in 25% (2/9) of patients, partial in 62% (6/9) and ineffective in 13% (1/9), with use of amiodarone;<br>Rosenbaum et al., 1987 reported that 92.6% (37/40) of patients showed a reduction in the number of ventricular tachycardia episodes with use of amiodarone and that 96.5% (40/41) of patients showed a reduction in the number of ventricular tachycardia episodes with use of flecainide.
**Ventricular premature beats**							
330 (7 observational studies and 1 randomized clinical trial)	serious [a]	not serious	not serious	not serious	very strong association	⨁⨁⨁◯ MODERATE	In IPD meta-analysis, amiodarone reduced ventricular premature beats in 93.1% (95%CI 82-97.4% in 24-hour Holter monitoring).<br>Belloti et al., 1983 reported that 83.33% (10/12) of patients showed a reduction in the number of ventricular premature beats.<br>Greco et al., 1980 reported that 35.30% (12/34) of patients had a great response, 58.81% (20/34) a good response, and 5.88% (2/34) a regular or no response, with use of amiodarone.<br>Patra et al., 1982 reported that 58.33% (70/120) of patients had a great response, 27.5% (33/120) a good response, 12.5% (15/120) a regular response, and 1.7% (2/120) no response, with use of amiodarone.<br>Vichi et al., 1984 reported that the number of premature beats decreased from 36.6 to 6.1% in 10 patients treated orally and 31% to 4.24% in 10 patients treated intravenously.<br>Carrasco et al., 1985 reported that antiarrhythmic effect was total in 67% (6/9) of patients, partial in 11% (1/9) and insignificant in 22% (2/9), with use of amiodarone.<br>Rosenbaum et al., 1987 reported that 90.7% (36/40) of patients showed a reduction in the number of ventricular premature beats with use of amiodarone and that 92.4% (38/41) of patients showed a reduction in the number of ventricular premature beats with use of flecainide.
**Ventricular couplets**							
119 (2 observational studies and 1 randomized clinical trial)	serious [a]	not serious	not serious	not serious	very strong association	⨁⨁⨁◯ MODERATE	In IPD meta-analysis, amiodarone reduced the incidence of ventricular couplets by 79.0% (RR 0.21, 95%CI 0.11-0.39 in 24-hour Holter monitoring).<br>Rosenbaum et al., 1987 reported that 95.2% (38/40) of patients showed a reduction in the number of ventricular couplets with use of amiodarone and that 92.5% (38/41) of patients showed a reduction in the number of ventricular couplets with use of flecainide.

Certainty assessment							Summary of findings
**Sudden death**							
119<br>(3 observational studies)	serious [a]	not serious	not serious	not serious	none	⊕◯◯◯<br>VERY LOW	Chiale et al., 1984 reported one case of sudden death during 5 months of treatment with amiodarone.<br>Greco et al., 1980 reported 5 cases of sudden death during treatment with amiodarone.<br>Scanavacca et al., 1990 reported 2 cases of sudden death during 27 months of treatment with amiodarone.

CI: Confidence interval; **RR:** Risk ratio

Explanations

a.The studies presented issues concerning lack of data about eligibility criteria, sample size, outcome assessor blinding, loss of follow-up and statistical analysis.
