# Supplementary material for: Amiodarone for arrhythmia in patients with Chagas disease: A systematic review and individual patient data meta-analysis
Source: PLoS Negl Trop Dis. 2018 Aug 20;12(8):e0006742. doi: 10.1371/journal.pntd.0006742 (PMC6130878; doi:10.1371/journal.pntd.0006742)

CORNEAL MICRODEPOSITS

Study	Events	Total	Events per 100 observations	Events	95%−CI	Weight
Chiale et al., 1984	24	24		100.00	[85.75; 100.00]	16.1%
Greco et al., 1980	48	48		100.00	[92.60; 100.00]	16.1%
Haedo et al., 1986	3	14		21.43	[4.66; 50.80]	22.2%
Rosenbaum et al.,1987	19	40		47.50	[31.51; 63.87]	24.0%
Scanavacca et al., 1990	2	35		5.71	[0.70; 19.16]	21.6%
**Random effects model**		**161**		**60.97**	**[18.81; 91.33]**	**100.0%**

Heterogeneity: $I^2 = 89\%$, $\tau^2 = 3.852$, $p < 0.01$

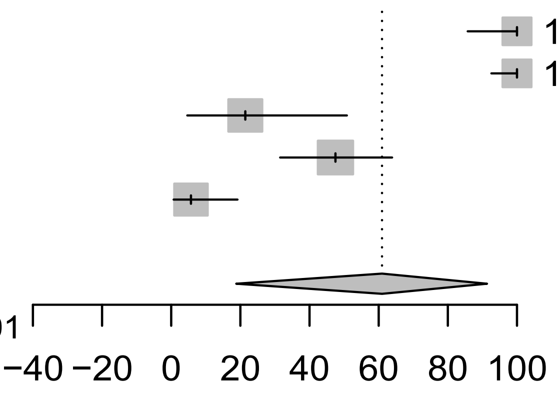

GASTROINTESTINAL EVENTS

Study	Events	Total	Events per 100 observations	Events	95%−CI	Weight
Carrasco et al., 1985	1	9		11.11	[0.28; 48.25]	19.4%
Chiale et al., 1984	2	24		8.33	[1.03; 27.00]	34.3%
Haedo et al., 1986	4	14		28.57	[8.39; 58.10]	46.3%
**Random effects model**		**47**		**16.11**	**[6.61; 34.23]**	**100.0%**

Heterogeneity: $I^2 = 26\%$, $\tau^2 = 0.2095$, $p = 0.26$

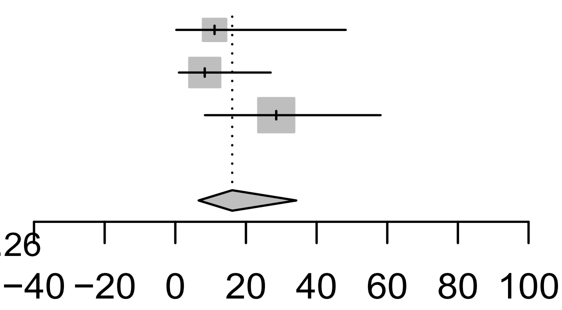

SYNUS BRADICARDIA

Study	Events	Total	Events per 100 observations	Events	95%−CI	Weight
Belotti et al., 1983	4	14		28.57	[8.39; 58.10]	19.0%
Carrasco et al., 1985	1	9		11.11	[0.28; 48.25]	14.4%
Chiale et al., 1984	2	24		8.33	[1.03; 27.00]	17.5%
Greco et al., 1980	1	48		2.08	[0.05; 11.07]	14.9%
Haedo et al., 1986	8	14		57.14	[28.86; 82.34]	19.4%
Scanavacca et al., 1990	1	35		2.86	[0.07; 14.92]	14.8%
**Random effects model**		**144**		**12.64**	**[3.68; 35.43]**	**100.0%**

Heterogeneity: $I^2 = 78\%$, $\tau^2 = 2.088$, $p < 0.01$

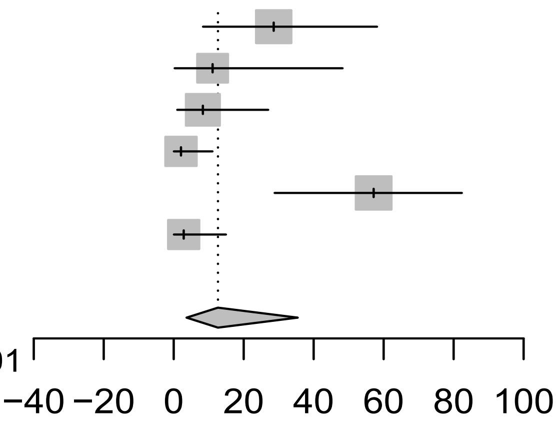

DERMATOLOGICAL EVENTS

Study	Events	Total	Events per 100 observations	Events	95%−CI	Weight
Chiale et al., 1984	2	24		8.33	[1.03; 27.00]	25.3%
Rosenbaum et al., 1987	2	40		5.00	[0.61; 16.92]	26.0%
Scanavacca et al., 1990	6	35		17.14	[6.56; 33.65]	48.7%
**Random effects model**		**99**		**10.54**	**[4.87; 21.32]**	**100.0%**

Heterogeneity: $I^2 = 30\%$, $\tau^2 = 0.17$, $p = 0.24$

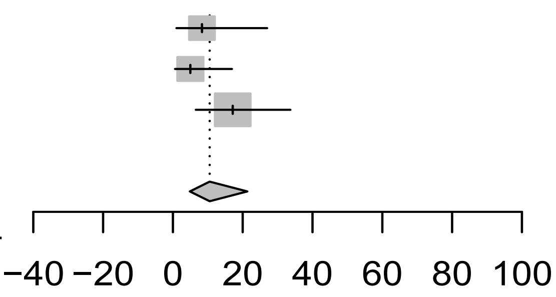

DRUG DISCONTINUATION

Study	Events	Total	Events per 100 observations	Events	95%−CI	Weight
Carrasco et al., 1985	1	9		11.11	[0.28; 48.25]	9.7%
Chiale et al., 1984	1	24		4.17	[0.11; 21.12]	10.5%
Greco et al., 1980	1	48		2.08	[0.05; 11.07]	10.7%
Rosenbaum et al., 1987	3	40		7.50	[1.57; 20.39]	30.3%
Scanavacca et al., 1990	4	35		11.43	[3.20; 26.74]	38.7%
**Random effects model**		**156**		**7.59**	**[4.12; 13.58]**	**100.0%**

Heterogeneity: $I^2 = 0\%$, $\tau^2 = 0$, $p = 0.55$

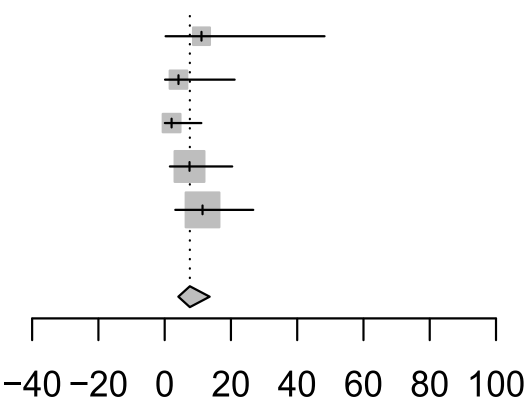

Supplement: S5 Appendix — (PDF) [file pntd.0006742.s006.pdf]
